# Circular RNA hsa_circ_0004812 impairs IFN-induced immune response by sponging miR-1287-5p to regulate FSTL1 in chronic hepatitis B

**DOI:** 10.1186/s12985-020-01314-0

**Published:** 2020-03-18

**Authors:** Liangdong Zhang, Zichao Wang

**Affiliations:** Second Department of Infectious Diseases, Cangzhou Central Hospital, Xinhua West Road, Cangzhou, 061001 Hebei Province China

**Keywords:** Chronic hepatitis B, Hepatitis B virus, Circular RNA, circ_0004812, miR-1287-5p

## Abstract

**Background:**

The present study aims to explore the functions of circular RNA hsa_circ_0004812 in chronic hepatitis B (CHB) and its underlying molecular mechanisms.

**Methods:**

The expression of circular RNA (circRNA)_0004812 was examined using qRT-PCR and Western blot in blood and liver tissues from CHB patients and healthy volunteers. In the in vitro study, the expression levels of circular RNA hsa_circ_0004812, miR-1287-5p, interferon (IFN)-α, IFN-β were determined using qRT-PCR and Western blotting in HBV-infected hepatoma cells, respectively. Luciferase and biotin pull-down assays were used to investigate the interactions between miR-1287-5p and circ_0004812.

**Results:**

Levels of circ_0004812 were upregulated in CHB patients and HBV-infected hepatoma cells. Knockdown of circ_0004812 increased the expression of IFN-α and IFN-β in HBV-infected Huh7 cells. MiR-1287-5p was identified as a target of circ_0004812 whose overexpression inhibited the expression of miR-1287-5p. Additionally, circ_0004812 promoted the expression of Follistatin-related protein (FSTL) 1 through inhibiting miR-1287-5p. Circ_0004812/miR-1287-5p/FSTL1 axis regulated HBV-induced immune suppression.

**Conclusion:**

Circ_0004812 was identified as a potential target for CHB infection. Circ_0004812 promoted the expression of FSTL1 by inhibiting miR-1287-5p.

## Background

Chronic hepatitis B virus (HBV) infection is one of the leading causes of liver fibrosis, cirrhosis, and liver cancer [1]. As a global health threat, more than 400 millions of people are infected with HBV worldwide [2–4]. Additionally, 90% of infants that are infected and 5–10% of healthy adults above 19 years old develop chronic hepatitis B (CHB) [2]. Antiviral therapy is recommended for the treatment of CHB by the American Association for the Study of Liver Diseases (AASLD) 2018 Hepatitis B Guidance [5]. Alternatively, interferon (IFN) therapy is also the first-line treatment of CHB [4, 6]. Once an individual is infected with HBV, acute liver damage occurs more easily and thereby leading to advanced liver diseases including liver fibrosis and cirrhosis [7]. Although the molecular mechanisms of CHB are not fully understood, the immune tolerance has been identified to play an important role in the development of CHB [1]. In the immune tolerance phase, high levels of HBV DNA copies are observed in the absence of strong immune response against the virus [3].

Circular RNA (circRNA) is one type of single-stranded RNAs and is characterized by a continuous loop in which the 3′ end is covalently linked with the 5′ end [8]. Unlike linear RNA, circRNA is stable RNA and produced by the circularization of exons [8, 9]. CircRNA can bind microRNA (miRNA) and act as a miRNA sponge to regulate gene expression [9]. As a novel type of non-coding RNA, circRNA has been identified to regulate some cellular events including cell growth and signaling transduction [10]. Additionally, it is reported that circRNA is associated with a variety of diseases including neurological diseases and cancers [10–12]. In 2018, Wang and colleagues reported the differentially expressed circRNAs in HBV-related hepatoma and identified that circRNA_101764 might play an important role in hepatoma [13]. In 2019, Yu and colleagues used plasma circRNA to diagnose HBV-related hepatocellular carcinoma [14]. These studies demonstrate that circRNA may play important roles in the pathogenesis and progression of CHB.

In 2018, Zhou and colleagues have reported that circ_0004812 is one of the top ten circRNAs significantly up-regulated in CHB compared to normal controls [15]. However, the underlying mechanisms of circ_0004812 in the CHB patients are still unclear. Therefore, the present study was designed to investigate the expression patterns of one circRNA, named circ_0004812, in the HBV-infect patients. First, we explored the effects of circ_0004812 in the IFN-induced immune response in CHB. Second, we investigated the miRNAs that were regulated by circ_0004812. Furthermore, we explored the mechanisms of circ_0004812 underlying HBV infection.

## Materials and methods

### Clinical specimens

Blood and liver tissue specimens were obtained from CHB patients (*n* = 25) and healthy volunteers (n = 25) enrolled in Cangzhou Central Hospital between 2017 and 2018. In brief, venous blood samples were obtained and immediately stored at − 80 °C for subsequent analysis. The liver biopsies were obtained from CHB patients, and non-infected (control) liver tissues were obtained from human liver grafts not suited for transplantation. All liver tissues were collected by using 16G disposable needles and then stored at − 80 °C. The protocol was approved by Cangzhou Central Hospital and all patients have read and signed informed consent.

### Cell lines and transfections

Huh7 and HepG2 cells were purchased from the Shanghai Cell Bank (Shanghai, China). HepG2.2.15 cells were established by transfection of HepG2 cells with plasmids containing the genomic sequence of HBV.

The cells were seeded into 24-well plates with a density of 1 × 10^5^ cells per well. When the cells reach 60–70% confluency, circRNAs (20 nM), siRNAs (20 nM), or miRNAs (20 nM) were transfected into the cells using Lipofectamine 2000 reagent (Invitrogen, Carlsbad, CA, USA) according to the manufacturer’s instructions. The sequences for siRNAs are shown as follows: si-Circ_0004812#1: 5′-GAG CTC ATG ACA GTG GAC AGT-3′; si-Circ_0004812#2: 5′-GAG AAG AGG AAC CTG GAG TTT-3′; si-negative control (NC): 5′-UUC UCC GAA CGU GUC ACG U-5′.

### Quantitative real-time reverse transcription polymerase chain reaction (qRT-PCR)

RNA was extracted from the cells using the RNA extraction kit, according to the manufacturer’s documentation. Reverse transcriptase was used in the RT reaction. The primers for the target genes are shown as follows: circ_0004812 forward: 5′- GCC TGC TAC CAC CA-3′, and reverse: 5′-TTG TCA CGC TCC CT-3′; IFN-α forward: 5′- GAA CTC TAC CAG CAG CT-3′ and reverse: 5′-CAG ATA GAG AGT GAT TC-3′; IFN-β forward: 5′-AAG GCC AAG GAG TAC AGT C, and reverse: 5′-AGT TTC GGA GGT AAC CTG-3′; miR-1287-5p forward: 5′-GTG CTG GAT CAG TGG TTC, and reverse: 5′-GTC CAG TTT TTT TTT TTT TTT GAC TC-3′; GAPDH forward: 5′-GAA GGT GAA GGT CGG AGT-3′, and reverse: 5′-GAA GAT GGT GAT GGG ATT TC-3′; U6 forward: 5′-CTC GCT TCG GCA GCA CA, and reverse: 5′-AAC GCT TCA CGA ATT TGC GT-3′. The melting curves were used to assess the accuracy. The expression of each gene was calculated using 2-^△△Ct^ method. The mRNA expression values of target genes were normalized to that of *U6* or *GAPDH*.

### Elisa

The levels of HBsAg, HBeAg, IFN-α, and IFN-β in the supernatant from the cell culture were determined using specific ELISA kits (R&D Biosystem, Minneapolis, MN, USA) according to the manufacturers’ documents.

### Western blot

The protein sample was extracted according to a previously reported method [16]. In brief, cold radioimmunoprecipitation buffer containing protease inhibitor was used to lyse the cells. After that, the extraction buffer was centrifuged at 12,000 *g* for 10 mins to remove the cell debris and other insoluble materials. The BCA protein assay kits were applied to qualify the concentrations of extracted proteins.

An equal amount of proteins was loaded and separated using the 10% sodium dodecyl sulfate gel. After that, the gel was transferred to a polyvinylidene fluoride membrane, which was blocked with 5% non-fat milk at room temperature for 2 h. Next, a primary antibody was used to incubate with the membrane at 4 °C overnight. Appropriate secondary antibodies conjugated with horse radish peroxidase were used and the imaging system was applied to qualify the expression of each target protein.

### Luciferase assays

Luciferase assays were applied to investigate the interactions between circ_0004812 and miR-1287-5p, and interactions between FSTL and miR-1287-5p, in the cells transfected with pHBV. In brief, when the cells reach 60–70% confluency, the cells were co-transfected with the luciferase reporter plasmids containing wild-type or mutant plasmids and miR-1287-5p or miRNA-negative control (NC). The activities of luciferase were determined 24 h after the transfection.

### Construction of mutant and overexpression cell lines

To construct circ_004812 mutant cell lines, the primers used for amplification of circ_004812 are shown as follows: circ_004812 forward: 5′-CAG TGG ACA GTG CCG TAC CGA AG-3′ and reverse: 5′-CTT CGG TAC GGC ACT GTC CAC TG-3′. After that, the PCR product was inserted into PLCDH-ciR. To construct circ_0076906 overexpression cell lines, the primers used for the amplification are as follows: circ_0076906 forward: 5′-CGG AAT TCT GAA ATA TGC TAT CTT ACA G AG CCT GGA CTT CAG CGT GG-3′ and reverse: 5′-CGG GAT CCT CAA GAA AAA ATA TAT TCA CTT GAT TTT CTT CTC CGT GAG CTG CT-3′. To ensure the overexpression cell lines were successfully constructed, we used qRT-PCR and Western blot to verify their expression.

### Biotin-coupled miRNA pulldown assay

Biotin-coupled miRNA pulldown assay was performed according to a previously reported method [17]. In brief, biotin-coupled miR-1287-5p was used to incubate with the cell lysates of pHBV-transfected Huh7 and HepG2.2.15 cells. After that, streptavidin-coupled agarose beads were applied to isolate the RNA-RNA complexes. All experimental procedures were performed under RNase free condition.

### Statistical analysis

All data were expressed as mean ± standard deviation (S.D). One-way analysis of variance with multiple comparisons and Student-Newman-Keuls test were performed. A *P*-value less than 0.05 was considered as a statistically significant difference between two groups.

## Results

### The levels of circ_0004812 were increased in CHB-infected patients

**Fig. 1:**
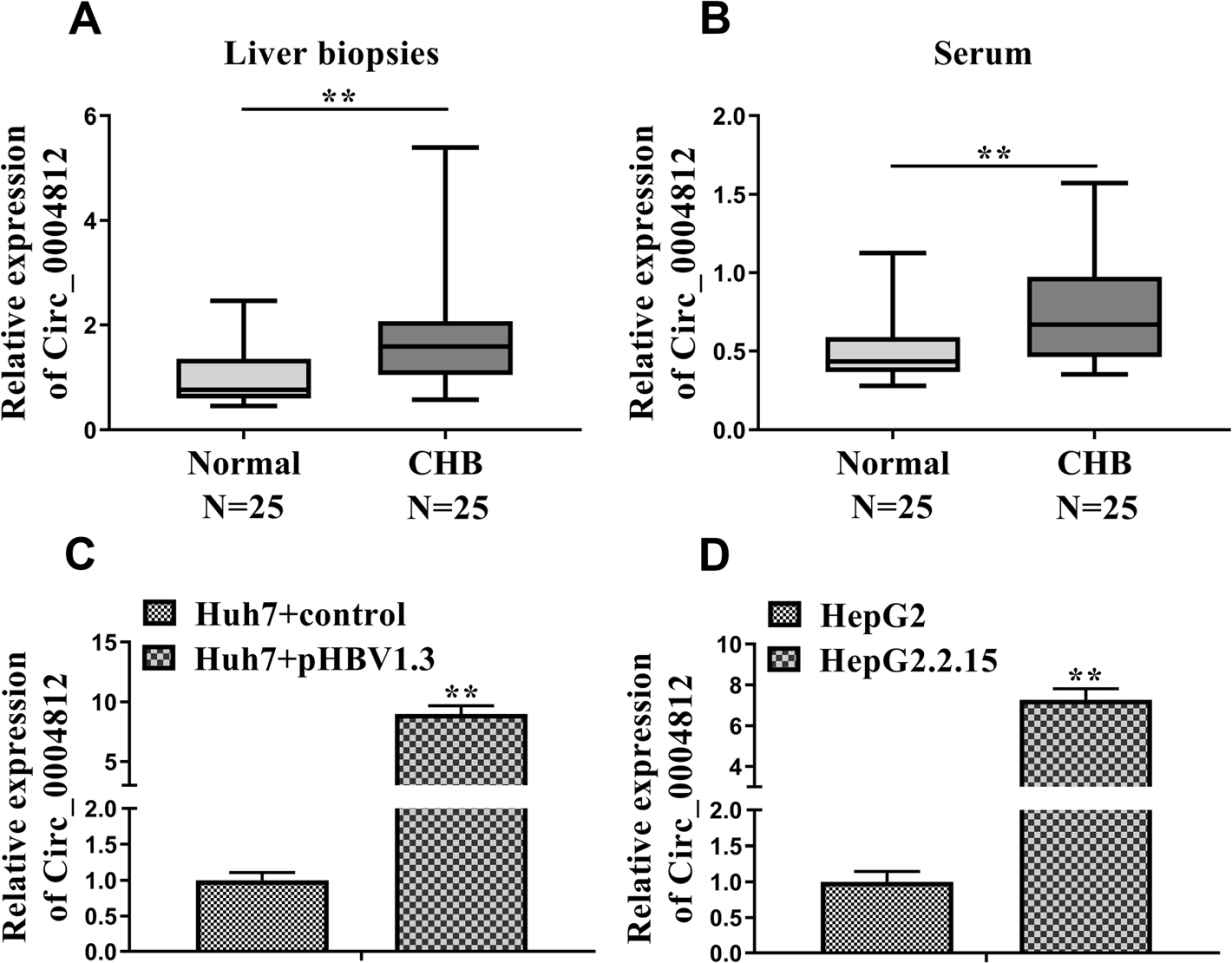
The levels of circ_0004812 were increased in chronic hepatitis B (CHB). **a**-**b** qRT-PCR was used to determine the relative expression of circ_0004812 in liver biopsies and serum of the CHB patients and healthy volunteers (*n* = 25). **c**-**d** The relative expression of circ_0004812 in hepatoma cells including Huh7 and HepG2 cells were determined. Additionally, pHBV1.3 was used to transfect Huh7 and HepG2 to establish HBV infected cells including Huh7 + pHBV1.3 cells and HepG2.2.15 cells and then qRT-PCR was used to determine the relative expression of circ_0004812 in those cells. Data were shown as mean ± S.D. **P < 0.01

First, we investigated the levels of circ_0004812 in liver tissue and blood from CHB patients and healthy volunteers. We observed that the levels of circ_0004812 in liver tissue (Fig. 1a) and blood (Fig. 1b) were significantly increased in the CHB-infected patients as compared with those in healthy volunteers (*P* < 0.01). We then constructed pHBV1.3-infected hepatoma cell lines Huh7 and HepG2. Interestingly, the results demonstrated that the level of circ_0004812 was significantly increased in the pHBV1.3-infected cells as compared with normal hepatoma cell line (Fig. 1c and d).

### Knockdown of circ_0004812 affected anti-HBV efficiency by regulating the levels of IFNs

**Fig. 2:**
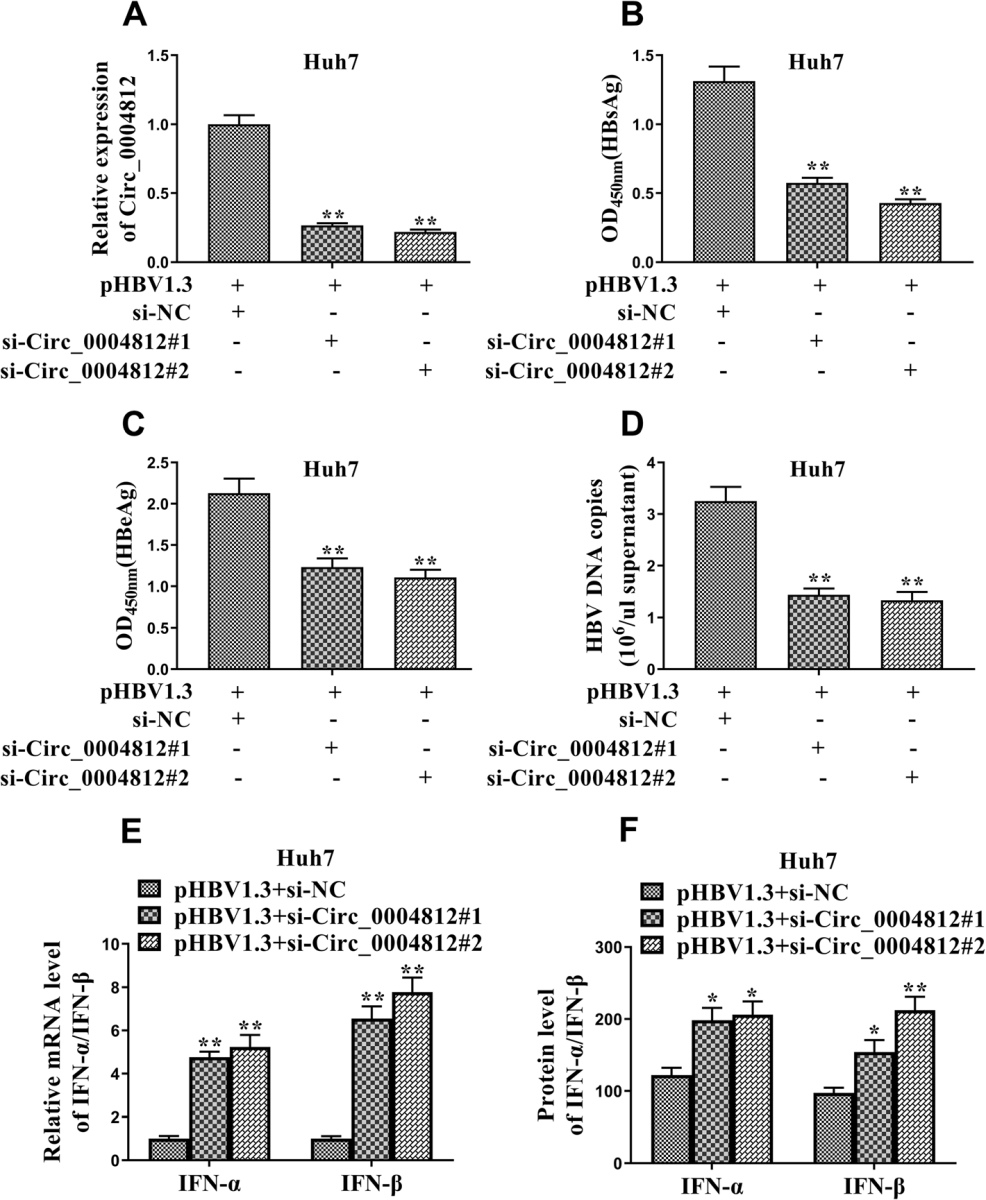
Knockdown of circ_0004812 affected anti-HBV efficiency by regulating the levels of IFNs. **a**-**f** pHBV1.3 was used to transfect the Huh7 cells. After that, the Huh7 cells were co-transfected with negative control siRNA (si-NC), circ_0004812 siRNAs including (si-circ_0004812#1, si-circ_0004812#2). **a** The relative expression of circ_0004812 were measured using qRT-PCR. **b**-**c** Levels of HBsAg and HBeAg were measured using specific ELISAs. **d** HBV DNA copies were determined using qRT-PCR. (E-F) The mRNA and protein levels of IFN-α and IFN-β were determined using qRT-PCR and Western blotting, respectively. Data were shown as mean ± S.D. *P < 0.05, **P < 0.01

We next verified the effects of circ_0004812 on the regulation of HBV infection in the cells. Two siRNAs targeting circ_0004812 were successfully applied to knockdown the expression of circ_0004812 in the Huh7 cells (Fig. 2a). After that, the levels of HBsAg, HBeAg, and HBV were examined in the pHBV1.3-transfected cells in the presence or absence of siRNAs targeting circ_0004812. The results demonstrated that levels of HBsAg and HBeAg were significantly decreased in the pHBV1.3-transfected cells co-transfected with circ_0004812 siRNAs, as compared to cells with only pHBV1.3 (Fig. 2b and c). Additionally, we also found that HBV DNA copies were significantly decreased in the pHBV1.3-transfected cells co-transfected with circ_0004812 siRNAs (Fig. 2d). We further determined the levels of IFN-α and IFN-β in these pHBV1.3-transfected cells. Interestingly, the results showed that the mRNA and protein levels of IFN-α and IFN-β were significantly increased in pHBV1.3-transfected cells co-transfected with circ_0004812 siRNAs (Fig. 2e and f). These results implied that the knockdown of circ_0004812 affected anti-HBV efficiency by regulating the levels of IFNs.

### Circ_0004812 regulated the levels of miR-1287-5p

**Fig. 3:**
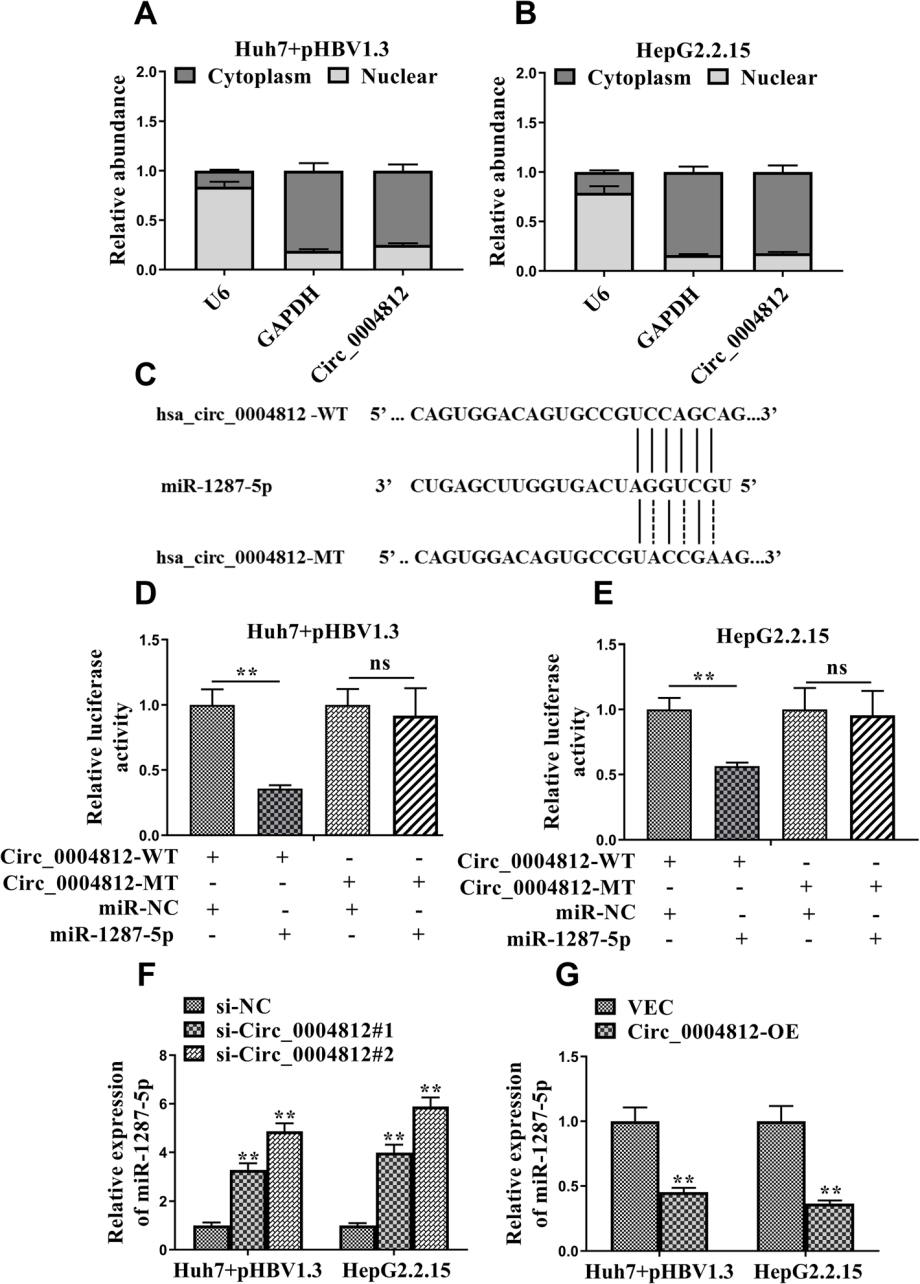
Circ_0004812 regulated the levels of miR-1287-5p. **a**-**b** qRT-PCR was applied to evaluate the abundance of circ_0004812 in the cytoplasm and nuclei in HepG2.2.15 cells and Huh7 cells that were transfected pHBV1.3. Two internal controls including cytoplasmic control (GADPH) and nuclear control (U6) were used. **c** The binding sites between miR-1287-5p and Circ_0004812 wild-type (circ_0004812-WT) and mutant (circ_0004812-MT) were shown. **d**-**e** Relative luciferase activities were determined in pHBV-transfected Huh7 or HepG2.2.15 cells co-transfected with circ_0004812-WT, circ_0004812-MT or psiCHECK2 empty vector and miR-1287-5p or miR-NC. **f** The relative expression of miR-1287-5p were determined using qRT-PCR in pHBV-transfected Huh7 and HepG2.2.15 cells that were co-transfected with negative control siRNA (si-NC), circ_0004812 siRNAs including (si-circ_0004812#1, si-Circ_0004812#2). Besides, (**g**) the relative expression of miR-1287-5p were also determined using qRT-PCR in pHBV-transfected Huh7 and HepG2.2.15 cells that were co-transfected with circ_0004812 overexpression plasmid (circ_0004812-OE) or empty vector (VEC). Data were shown as mean ± S.D. *P < 0.05, **P < 0.01, and ns indicates not significant

We explored the distributions of circ_0004812 in the cells. The results showed that circ_0004812 was abundant in the cytoplasm (Fig. 3a and b). We then predict the targets of circ_0004812 using the Circular RNA Interactome. As shown in Fig. 3c, miR-1287-5p was identified as a target of circ_0004812. To confirm their interactions, the luciferase assay was performed. The results demonstrated that the relative activities of luciferase were significantly decreased in the cells co-transfected with circ_0004812 wide type (WT) and miR-1287-5p, as compared with those in the cells co-transfected with circ_0004812 wide type (WT) and miR-NC (Fig. 3d and e). We further determined the expression of miR-1287-5p in the pHBV1.3-transfected cells. The results demonstrated that the expression of miR-1287-5p was significantly increased in the circ_0004812 siRNA transfected cells (Fig. 3f). Additionally, we noticed that the expression of miR-1287-5p was significantly decreased in the circ_0004812 overexpressing cells (Fig. 3g). These results supported that circ_0004812 negatively regulated the levels of miR-1287-5p in the pHBV1.3-transfected cells.

### Circ_0004812 promoted the expression of FSTL1 through inhibition of miR-1287-5p

**Fig. 4:**
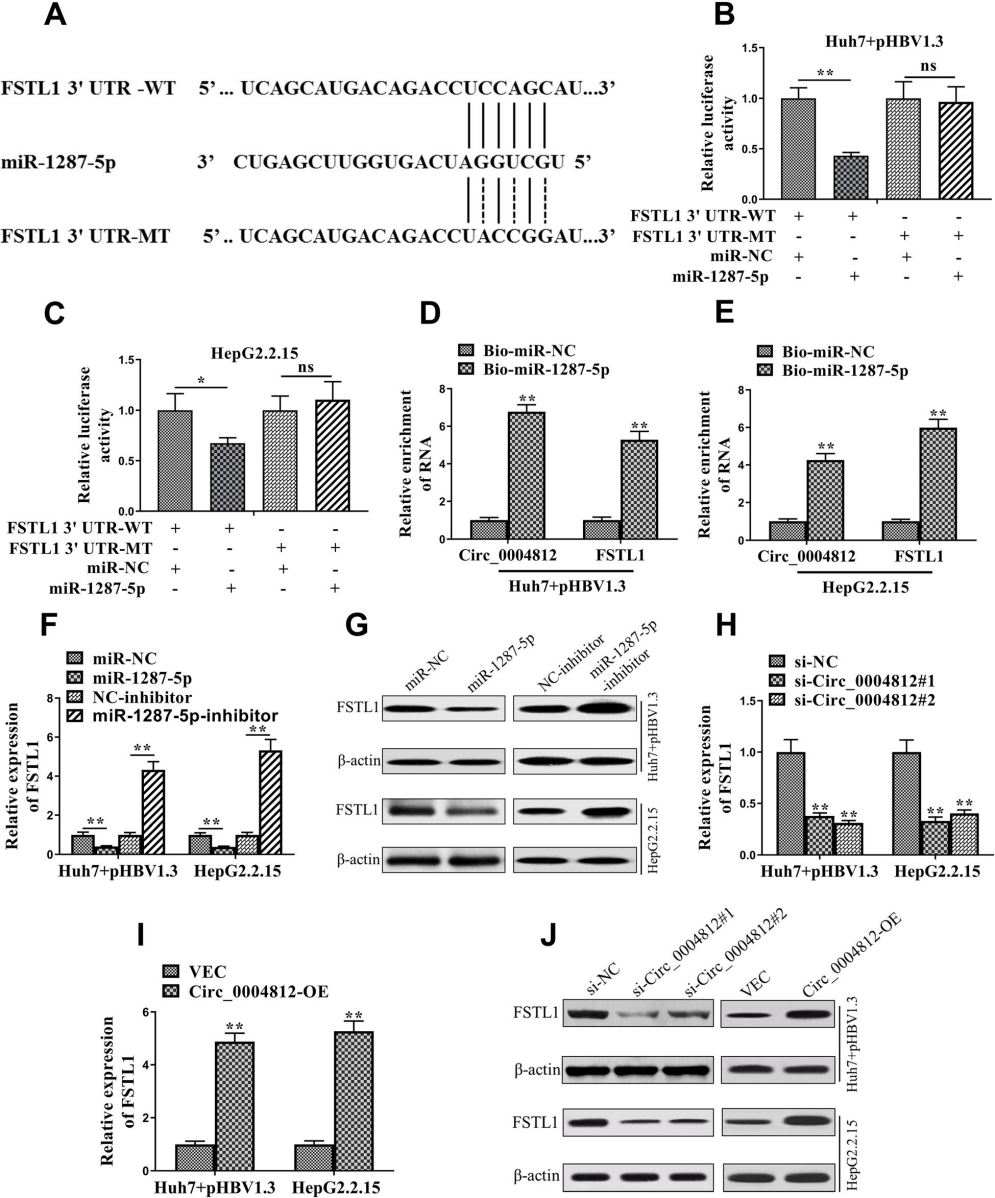
Circ_0004812 promoted the expression of FSTL1 through inhibition of miR-1287-5p. **a** The binding sites between miR-1287-5p, FSTL1 3’UTR-WT, and FSTL1 3’UTR-MT were predicted and showed. **b**-**c** The relative luciferase activities were determined in the pHBV-transfected Huh7 and HepG2.2.15 cells that were co-transfected with FSTL1 3’UTR-WT plus miR-NC, FSTL1 3’UTR-WT plus miR-1287-5p, FSTL1 3’UTR-WT plus miR-NC, or FSTL1 3’UTR-MT plus miR-1287-5p. **d**-**e** RNA pull-down assays were used to detect the relative enrichment of RNA including circ_0004812 and FSTL1 in pHBV-transfected Huh7 and HepG2.2.15 cells. **f**-**g** The relative mRNA and protein levels of FSTL1 were determined using qRT-PCR and Western blotting, respectively, in pHBV-transfected Huh7 and HepG2.2.15 cells that were co-transfected with miR-1287-5p mimics and miR-1287-5p inhibitor or the negative controls. **h**-**j** The relative mRNA and protein levels of FSTL1 were determined using qRT-PCR and Western blotting, respectively, in pHBV-transfected Huh7 and HepG2.2.15 cells that were co-transfected with Circ_0004812 siRNAs including si-Circ_0004812#1 and si-Circ_0004812#2) or the negative control siRNA (si-NC), or plasmids containing sequence of Circ_0004812 or empty vector (VEC). Data were shown as mean ± S.D. *P < 0.05, **P < 0.01, and ns indicates not significant

We next explored the potential targets of miR-1287-5p. By using Targetscan (http://www.targetscan.org/vert_71/), FSTL1 was identified as a target of miR-1287-5p and the binding sites between them were shown in Fig. 4a. Next, the luciferase assay was performed to verify the interactions between miR-1287-5p and FSTL1. The results demonstrated that relative activity of luciferase was significantly decreased in the pHBV1.3-transfected Huh7 cells co-transfected with FSTL1 3’UTR-WT and miR-1287-5p, as compared with those in the cells co-transfected with FSTL1 3’UTR-WT and miR-NC (Fig. 4b). Similarly, we also observed this phenomenon in the HepG2.2.15 cells (Fig. 4c). We next applied biotin pull-down assay to verify the interactions among miR-1287-5p, FSTL1, and circ_0004812. The results showed that both FSTL1 and circ_0004812 were enriched in biotin-coupled miR-1287-5p pull-down complexes (Fig. 4d and e).

We next investigated the effects of miR-1287-5p on the expression of FSTL1. The results demonstrated that the mRNA and protein levels of FSTL1 were decreased in the miR-1287-5p transfected cells, whereas increased in the miR-1287-5p inhibitor-treated cells (Fig. 4f and g). Furthermore, the effects of circ_0004812 on the expression of FSTL1 were explored. The results showed that the mRNA and protein levels of FSTL1 were decreased in the siRNA circ_0004812 transfected cells, whereas increased in the circ_0004812 overexpressing cells (Fig. 4h-j).

### The expression patterns of miR-1287-5p and FSTL1 in HBV-transfected cells

**Fig. 5:**
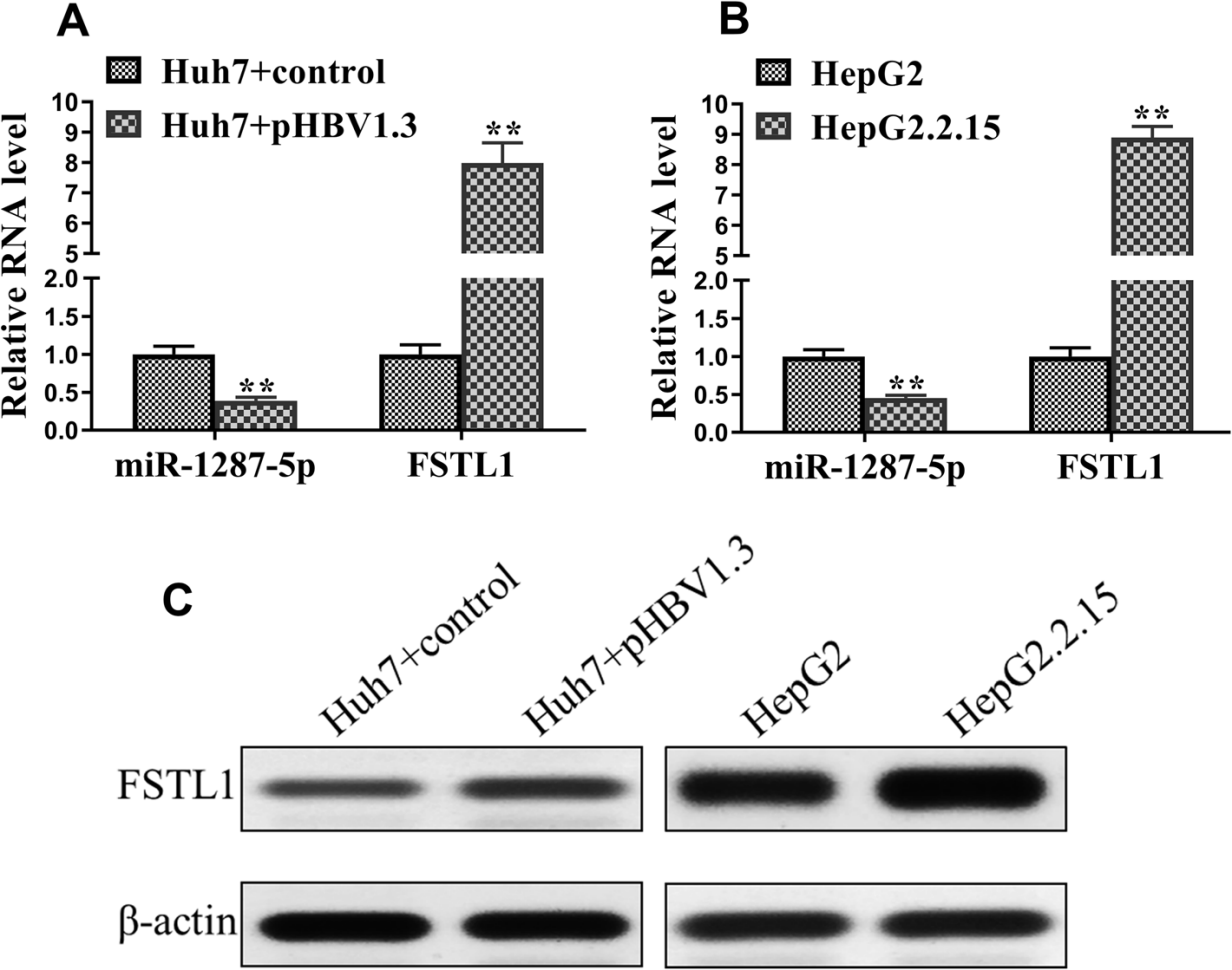
The expression patterns of miR-1287-5p and FSTL1 in HBV-transfected cells. **a**-**c** The mRNA and protein levels of miR-1287-5p and FSTL1 were determined using qRT-RCR and Western blotting, respectively, in huh7 cells plus control, Huh7 cells plus pHBV1.3, HepG2 cells, and HepG2.2.15 cells. *P < 0.05; **P < 0.01

The expression patterns of miR-1287-5p and FSTL1 were then investigated. The results showed the mRNA levels of miR-1287-5p were decreased in both of the HBV-infected hepatoma cell lines Huh7 and HepG2 (Fig. 5a and b). However, the mRNA levels of FSTL1 were significantly increased in the HBV-infected hepatoma cells as compared with those in the normal hepatoma cells (Fig. 5a and b). Similarly, the level of miR-1287-5p was decreased and the protein level of FSTL1 was increased in the HBV-infected hepatoma cell lines as compared with those in the normal hepatoma cell lines (Fig. 5c).

### Circ_0004812/miR-1287-5p/FSTL1 axis regulated HBV-induced immune suppression

**Fig. 6:**
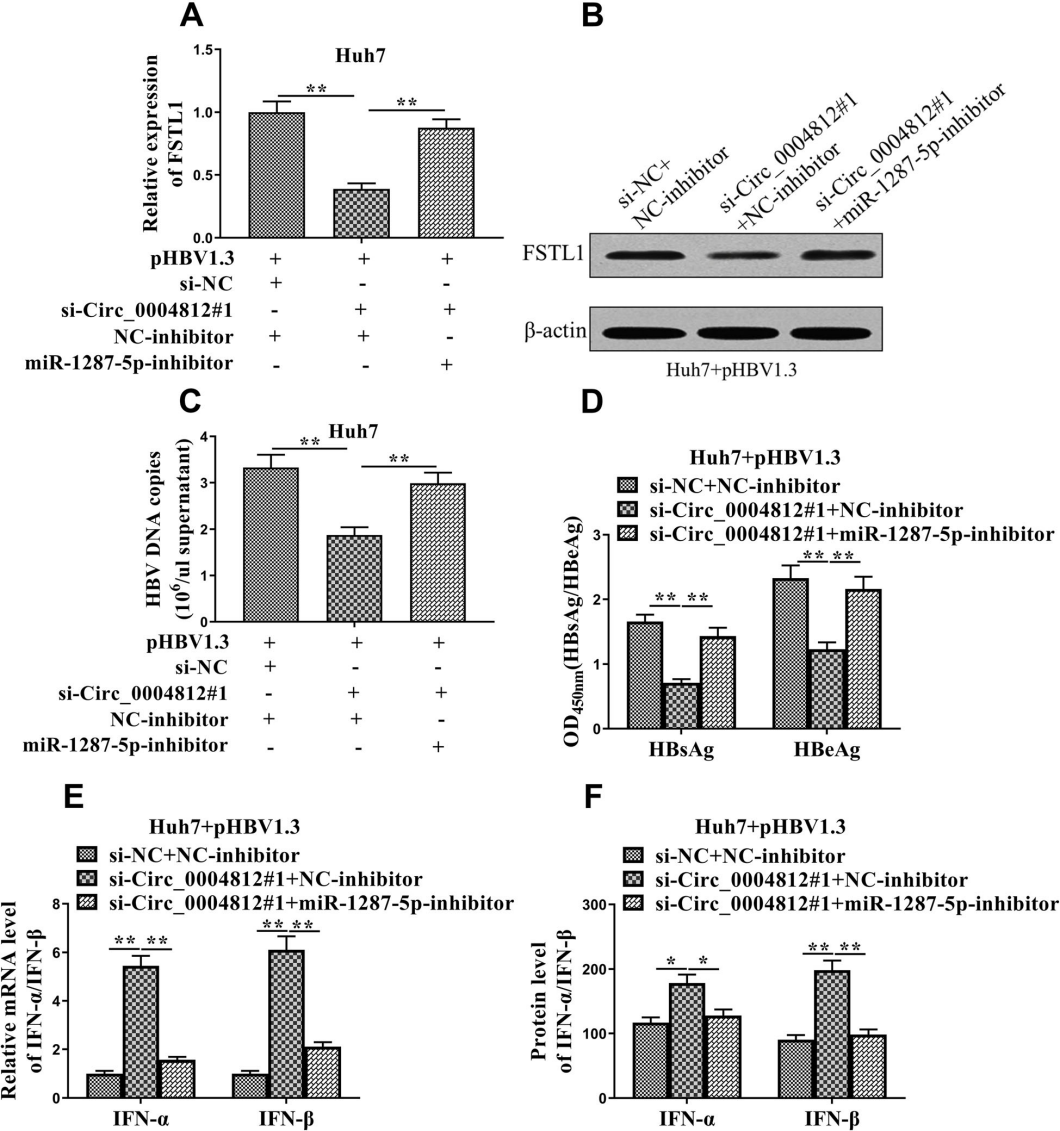
Circ_0004812/miR-1287-5p/FSTL1 axis regulated HBV-induced immune suppression. **a**-**f** pHBV1.3 was used to transfect the Huh7 cells. After that, the pHBV1.3-transfected Huh7 cells were co-transfected with si-NC plus NC-inhibitor, si-Circ_0004812#1 plus NC-inhibitor, or si-circ_0004812#1 plus miR-1287-5p-inhibitor. Next, (**b**-**c**) the mRNA and protein levels of FSTL1 were determined using qRT-RCR and Western blotting, respectively. **c** HBV DNA copies were determined using qRT-PCR. **d** Levels of HBsAg and HBeAg were measured using specific ELISAs and the ration of HBsAg to HBeAg was calculated. **e**-**f** The mRNA and protein levels of IFN-α and IFN-β were determined using qRT-PCR and Western blotting, respectively. The ratio of IFN-α to IFN-β were calculated. Data were shown as mean ± S.D. *P < 0.05, **P < 0.01

We explored the effects of circ_0004812/miR-1287-5p/FSTL1 axis on the regulation of HBV infection. First, circ_0004812 and miR-1287-5p were inhibited in the HBV-infected Huh7 cells. The results showed that the levels of FSTL1 were decreased in the circ_0004812 knockdown cells, and were recovered by miR-1287-5p inhibitor (Fig. 6a and b). Next, we examined the levels of HBsAg, HBeAg, HBV DNA copies, IFN-α, and IFN-β in those cells. As shown in Fig. 6c and d, the levels of HBV DNA copies, and the ratio of HBsAg to HBeAg were reduced in the circ_0004812 knockdown cells. However, the levels of HBV DNA copies and the ratio of HBsAg to HBeAg were increased in the circ_0004812 knockdown cells treated with miR-1287-5p inhibitor. Additionally, the mRNA and protein levels of IFN-α and IFN-β were increased in the circ_0004812 knockdown cells, whereas increased IFN-α and IFN-β were observed in the circ_0004812 knockdown cells treated with miR-1287-5p inhibitor (Fig. 6e and f). These results supported that circ_0004812/miR-1287-5p/FSTL1 axis regulated HBV-induced immune suppression.

## Discussion

In the present study, we identified a circRNA named circ_0004812 to be upregulated in the CHB patients as well as HBV-infected hepatoma cell lines Huh7 and HepG2. We found that circ_0004812 regulated anti-HBV efficiency by regulating the levels of IFNs. Additionally, miR-1287-5p was identified as a target of circ_0004812. By regulating miR-1287-5p, circ_0004812 promoted the expression of FSTL1. Our results demonstrated that circ_0004812/miR-1287-5p/FSTL1 axis regulated HBV-induced immune suppression.

CircRNA is more stable than linear RNA, therefore could be more suitable as a biomarker for liver diseases [8, 11, 18]. In recent years, circRNAs have been revealed to play important roles in the occurrence and development of CHB [19]. For instance, Wong and colleagues have demonstrated that the HBV circRNA is predominant in the liver of CHB patients [20]. In 2018, Wang and colleagues reported the differentially expressed circRNAs in HBV-related hepatoma and identified that circRNA_101764 might play an important role in hepatoma [13]. In 2019, Yu and colleagues used plasma circRNA to diagnose HBV-related hepatocellular carcinoma [14]. These studies highlighted that circRNA might be used as a therapeutic biomarker in the development of CHB. In 2018, Zhou and colleagues have reported that circ_0004812 is one of the top ten circRNAs significantly up-regulated in CHB compared to normal controls [15] Interestingly, we also identified that circ_0004812 was upregulated in CHB patients as well as HBV-infected hepatoma cell lines.

We further knocked down the level of circ_0004812 in the HBV-infected hepatoma cells, then explored the anti-HBV efficiency of those cells. Interestingly, levels of HBsAg, HBeAg, and HBV DNA copies were significantly decreased in the HBV-infected cells after circ_0004812 knockdown. It is known that the levels of HBsAg, HBeAg, and HBV DNA copies are prognostic biomarkers of CHB [21]. The decrease in HBsAg and HBeAg is an ideal endpoint in CHB after anti-viral therapy [22]. Not only HBV DNA copies act as a prognostic biomarker, but also the levels of HBV DNA copies are associated with inflammation and fibrosis in the CHB patients [23]. Moreover, in the present study, we also determined the levels of IFNs including IFN-α and IFN-β in the HBV-infected cells co-transfected with circ_0004812 siRNAs. Both IFN-α and IFN-β have been reported to inhibit HBV replication [24]. Our results demonstrated that the mRNA and protein levels of IFN-α and IFN-β were significantly increased after circ_0004812 knockdown in the HBV-infected cells. These results suggest that circ_0004812 affects anti-viral efficiency by regulating IFNs levels.

It is known that circRNA can act as a microRNA sponge, or bind with proteins to form a scaffold, thereby regulating gene expression [8]. In this study, we first identified miR-1287-5p as the target of circ_0004812. MiR-1287-5p has been revealed to play important roles in a variety of cancers including colorectal cancer, desmoids cancer, and breast cancer. For instance, a previous study has revealed the role of miR-1287-5p in triple-negative breast cancer through the regulation of phosphoinositide 3-kinase CB [25]. However, the roles of miR-1287-5p in the development of CHB are unclear. In the present study, for the first time, we identified miR-1287-5p as a target of circ_0004812. Our results demonstrate that circ_0004812 negatively regulates miR-1287-5p level in HBV infected cells.

Furthermore, we revealed that, by the inhibition of miR-1287-5p, circ_0004812 promoted the expression of FSTL1. FSTL1 has been reported to play crucial roles in the development of organs including the central nervous system, ureter, and lung [26, 27]. Besides, FSTL1 is also associated with rheumatoid arthritis and cardiovascular diseases [26, 28]. More importantly, FSTL1 has been implicated in fibrosis and tumorigenesis [26]. In the present study, we observed that the expression of FSTL1 was increased in the HBV-infected hepatoma cell lines as compared with those in the normal hepatoma cell lines. These results imply that HBV infection may promote FSTL1 expression. However, the underlying mechanism should be investigated in future study. Interestingly, we found that circ_0004812 promoted the expression of FSTL1 through inhibiting miR-1287-5p. The results showed that the expression of FSTL1 was decreased after circ_0004812 knockdown in the HBV-infected cells, whereas the expression of FSTL1 was increased in the circ_0004812 overexpressing cells. Moreover, we determined the effects of circ_0004812/miR-1287-5p/FSTL1 axis on the HBV-induced immune suppression. The results demonstrated that the HBV DNA copies and the ratio of HBsAg to HBeAg were recovered in the circ_0004812 knockdown cells treated with miR-1287-5p inhibitor. Additionally, miR-1287-5p inhibitor increased the expression levels of IFN-α and IFN-β in the circ_0004812 knockdown cells. Overall, these results demonstrate that the circ_0004812/miR-1287-5p/FSTL1 axis regulates HBV-induced immune suppression and may be used as a potential therapeutic target for the treatment of CHB.

## Conclusions

We identified that circ_0004812 was upregulated in the CHB patients. The knockdown of circ_0004812 affected anti-HBV efficiency by regulating the levels of IFNs. Additionally, we identified miR-1287-5p as a target of circ_0004812 and the overexpression of circ_0004812 inhibited the expression of miR-1287-5p. Furthermore, circ_0004812 promoted the expression of FSTL1 through inhibiting miR-1287-5p. These results demonstrated that circ_0004812 might be as a potential target for treatment of HBV infection.

## Data Availability

The data could be obtained upon request to the corresponding authors.

## Abbreviations

circ: Circular RNA
CHB: Chronic hepatitis B
FSTL: Follistatin-related protein
IFN: Interferon
HBV: Chronic hepatitis B virus
AASLD: American association for the study of liver diseases
miRNA: microRNA
NC: Negative control
S.D: Standard deviation
